# Impact of Liver Cirrhosis on Pregnancy Outcomes: A Retrospective Cohort Study from the TriNetX Global Collaborative Network

**DOI:** 10.3390/medicina62030591

**Published:** 2026-03-20

**Authors:** Ji-Ze Hsu, Dah-Ching Ding

**Affiliations:** 1 Department of Medical Research, Hualien Tzu Chi Hospital, Buddhist Tzu Chi Medical Foundation, Tzu Chi University, Hualien 970, Taiwan; jize930@gmail.com; 2 Department of Obstetrics and Gynecology, Hualien Tzu Chi Hospital, Buddhist Tzu Chi Medical Foundation, Tzu Chi University, Hualien 970, Taiwan; 3 Institute of Medical Sciences, Tzu Chi University, Hualien 970, Taiwan

**Keywords:** liver cirrhosis, pregnancy outcomes, maternal complications, fetal complications, propensity score matching

## Abstract

*Background and Objectives:* To evaluate the impact of liver cirrhosis on pregnancy outcomes using a large-scale, propensity score-matched cohort, with adjustment for numerous confounding variables. *Materials and Methods:* From a total of 3,701,876 pregnancies (women aged 18–49) from 1 January 2010, to 31 December 2024, after propensity score matching, 2498 pregnancies with cirrhosis and 2498 pregnancies without cirrhosis in TrinetX database were included in our analysis. To adjust for potential confounding, pregnancies in the cirrhosis group were matched 1:1 to those without cirrhosis using propensity scores derived from demographic, lifestyle, comorbidity, and laboratory characteristics. Relative risks (RRs), risk differences (RDs), and corresponding 95% confidence intervals (CIs) were calculated for pregnancy-related outcomes. Subgroup analyses stratified by maternal age were further performed to assess potential effect modification. Main outcomes included Gestational diabetes mellitus, preeclampsia, premature rupture membranes, preterm birth, miscarriage, stillbirth, placental abruption, dystocia, postpartum hemorrhagia, and cesarean delivery. *Results:* After matching, 2485 women were included in each group, with well-balanced baseline characteristics. Compared with women without cirrhosis, those with cirrhosis had a higher risk of pregnancy-related outcomes, including gestational diabetes mellitus (15.5% vs. 11.9%; RR = 1.30; 95% CI, 1.13–1.50, *p* < 0.001), preeclampsia (8.6% vs. 5.7%; RR = 1.52; 95% CI, 1.24–1.87, *p* < 0.001), and preterm birth (9.0% vs. 4.9%; RR = 1.85; 95% CI, 1.49–2.29, *p* < 0.001). Cirrhosis during pregnancy was also associated with a higher risk of miscarriage (6.6% vs. 4.8%), stillbirth (1.3% vs. 0.5%), placental abruption (1.8% vs. 0.8%), postpartum hemorrhage (6.9% vs. 4.3%), and cesarean delivery (20% vs. 17.2%). The limitations include the lack of detailed data on cirrhosis severity. *Conclusions:* Pregnancy with liver cirrhosis is associated with increased risks of diverse maternal and neonatal complications. Our findings highlight the importance of multidisciplinary management and individualized care planning in order to reduce adverse outcomes.

## 1. Introduction

Liver cirrhosis in pregnancy is rare, with an estimated prevalence of 1 in 3333 to 1 in 4500 pregnancies [1]. The prevalence of pregnancy with liver cirrhosis is influenced by the underlying causes, including autoimmune and viral hepatitis, as well as alcoholic and nonalcoholic fatty liver disease [1]. A university hospital study reported that the incidence of pregnancy with cirrhosis was 0.06% [2]. Despite its rarity, pregnancy with cirrhosis poses significant risks for both maternal and fetal health. Specifically, it increases the risk of adverse outcomes such as cesarean section, preterm birth, and postpartum hemorrhage [2]. Further, it is associated with a high risk of preterm birth, with an incidence rate of 46.51% [2]. Additionally, pregnancy with cirrhosis is associated with an increased incidence of severe maternal adverse events, including cesarean sections and intensive care unit admission [3].

Pregnancy in women with preexisting liver disease represents a complex clinical scenario that has become increasingly encountered in modern obstetric and hepatology practice. Chronic liver conditions—ranging from cirrhosis and portal hypertension to viral hepatitis, autoimmune hepatitis (AIH), metabolic dysfunction-associated steatotic liver disease (MASLD), and rarer entities such as Wilson’s disease and Budd-Chiari syndrome—each carry distinct risks for both mother and fetus [4,5]. Historically, advanced liver disease was considered incompatible with successful pregnancy due to profound hormonal dysregulation and subfertility; however, improvements in the medical management of underlying hepatic conditions, alongside advances in assisted reproductive technologies, have made pregnancy an achievable goal for a growing number of affected women [6,7].

The presence of cirrhosis and portal hypertension substantially elevates the risk of adverse maternal and fetal outcomes. Pregnant women with cirrhosis face significantly higher rates of variceal hemorrhage, hepatic decompensation, preeclampsia, preterm birth, cesarean delivery, and postpartum hemorrhage compared to the general obstetric population [1,8]. Fetal complications, including low birth weight, small-for-gestational-age neonates, and perinatal mortality, are similarly increased [2,3]. Disease severity, as measured by scoring systems such as the Child-Turcotte-Pugh (CTP) score and the albumin-bilirubin (ALBI) score, has been identified as a key predictor of these adverse events, underscoring the importance of thorough pre-pregnancy risk stratification [8,9]. Notably, women with compensated cirrhosis appear to face a substantially more favorable trajectory than those with decompensated disease, and pregnancy itself does not appear to independently accelerate liver disease progression in this group [10].

Given the multifaceted risks involved, the management of pregnancy complicated by preexisting liver disease requires a coordinated, multidisciplinary approach encompassing hepatology, obstetrics, and neonatology. Pre-conception counseling is paramount to ensure that underlying liver disease is optimally controlled prior to conception, as demonstrated in AIH, where incomplete biochemical response at the time of conception significantly increases the risk of gestational relapse, hypertensive disorders, and intrahepatic cholestasis of pregnancy [11,12]. Liver transplant recipients constitute a unique and growing subgroup in whom pregnancy is feasible but carries elevated risks of preterm delivery, gestational diabetes, and—in a minority—graft rejection, necessitating vigilant monitoring throughout gestation [13,14]. Across all etiologies of chronic liver disease, early identification of at-risk patients, individualized management of hepatic and obstetric complications, and close surveillance through delivery and the postpartum period remain the cornerstones of optimizing outcomes for both mother and child [4,5].

Liver cirrhosis may adversely affect pregnancy through multiple interrelated mechanisms, including portal hypertension, altered systemic hemodynamics, coagulopathy, chronic inflammation, and impaired glucose and lipid metabolism [15]. These pathophysiological changes can compromise placental perfusion and maternal–fetal exchange, thereby increasing the risk of hypertensive disorders, metabolic complications, hemorrhage, preterm birth, and other adverse obstetric outcomes [16].

Available evidence on pregnancy outcomes in women with liver cirrhosis is limited and inconsistent [6]. Most prior studies are small, single-center, or registry-based, often lacking adequate adjustment for confounding factors and rarely incorporating age-stratified analyses [17]. Consequently, the magnitude of cirrhosis-associated obstetric risk and the extent to which maternal age modifies these associations remain incompletely understood [18].

Despite increasing evidence from registry-based studies and meta-analyses, several important gaps remain [1,8]. Many previous studies were derived from single-country administrative databases and provided limited evaluation of potential age-related differences in risk [2,19]. Furthermore, the clinical implications of age-specific associations have not been well characterized. To address these limitations, we conducted a large-scale cohort study using the TriNetX global research network, which integrates data from multiple healthcare organizations. By performing age-stratified analyses, this study aims to better characterize the potential variation in risk across different age groups and to provide clinically relevant insights that may assist physicians in risk stratification and patient counseling.

## 2. Materials and Methods

### 2.1. Study Design and Data Sources

This retrospective cohort study was conducted using data from the Global Collaborative Network of TriNetX (Cambridge, MA, USA), a global health research platform that provides access to de-identified, real-time electronic health record (EHR) data from more than 200 million patients across approximately 170 healthcare organizations (HCOs) in over 20 countries. Clinical information within the platform is standardized and coded using internationally recognized terminologies, including the International Classification of Diseases, Tenth Revision, Clinical Modification (ICD-10-CM) for diagnoses, the International Classification of Diseases, Tenth Revision, Procedure Coding System (ICD-10-PCS) and Current Procedural Terminology (CPT) for procedures, as well as selected laboratory test results [20,21].

This study was approved by the Research Ethics Committee of Hualien Tzu Chi Hospital (IRB No. IRB114-103-C). The requirement for informed consent was waived because of the retrospective study design and the use of de-identified data.

### 2.2. Study Population and Exposure

**Figure 1:**
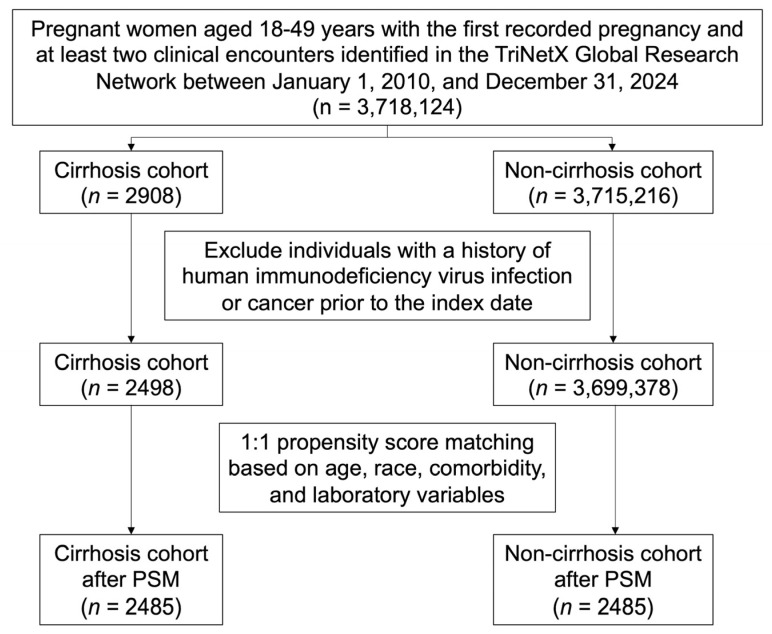
Flow diagram of cohort selection and propensity score matching from the TriNetX database. PSM: propensity score matching.

The study population comprised women aged 18–49 years who had a recorded pregnancy event and at least two clinical encounters between 1 January 2010, and 31 December 2024, within the TriNetX database. The index date was defined as the date of the first recorded pregnancy. Pregnancy was identified based on relevant ICD-10-CM codes, including Z33, Z34, and Z3A. Participants were categorized according to the presence of cirrhosis prior to the index date. Women with a documented diagnosis of cirrhosis before their first pregnancy, defined by ICD-10-CM codes K70.3 or K74, were assigned to the cirrhosis group, whereas those without any cirrhosis diagnosis before the index date were assigned to the non-cirrhosis group. To reduce potential confounding, the exclusion criteria included a diagnosis of human immunodeficiency virus infection (ICD-10-CM: B20 or Z21) or malignant neoplasms (ICD-10-CM: C00–D49) prior to the index date. Because the TriNetX database is derived from electronic health records and coded diagnoses, detailed indicators of cirrhosis severity, including Child–Pugh classification, MELD score, and specific complications such as portal hypertension, ascites, varices, or hepatic encephalopathy, were not consistently available. Therefore, stratification according to cirrhosis severity could not be performed. The stepwise exclusion process is illustrated in Figure 1.

### 2.3. Outcome Measures

Pregnancy onset in this study was approximated using the first recorded pregnancy-related diagnostic codes (ICD-10-CM: Z33, Z34, and Z3A) as the index event. The primary outcomes were pregnancy-related outcomes occurring within 1 year after the index date. This follow-up window (1 year) was selected to cover a typical gestational period (approximately 280 days) as well as a postpartum period of 42 days. The use of this broader time window was intended to minimize the risk of missing pregnancy-related complications recorded during routine clinical care. The primary outcomes included gestational diabetes mellitus (ICD-10-CM: O24), preeclampsia (ICD-10-CM: O14), premature rupture of membranes (ICD-10-CM: O42), preterm birth (ICD-10-CM: O60), miscarriage (ICD-10-CM: O03, O04, and O07), stillbirth (ICD-10-CM: O36.4), placental abruption (ICD-10-CM: O45), dystocia (ICD-10-CM: O62–O66), and postpartum hemorrhage (ICD-10-CM: O72). Cesarean delivery was identified using ICD-10-CM code O82, ICD-10-PCS codes 10D00Z0, 10D00Z1, and 10D00Z2, as well as CPT codes 1014218, 1008991, 59515, and 59620.

### 2.4. Covariates and Confounders

Baseline characteristics were defined as measurements or diagnoses documented within 1 year prior to the index date. Demographic variables included age at the index date and race. Lifestyle factors included nicotine dependence (ICD-10-CM: F17) and alcohol-related disorders (ICD-10-CM: F10). Comorbidities included diabetes mellitus (ICD-10-CM: E08–E13), hypertension (ICD-10-CM: I10), hyperlipidemia (ICD-10-CM: E78), fatty liver disease (ICD-10-CM: K76.0), toxic liver disease (ICD-10-CM: K71), hepatic failure (ICD-10-CM: K72), chronic hepatitis (ICD-10-CM: K73), other inflammatory liver diseases (ICD-10-CM: K75), other diseases of liver (ICD-10-CM: K76), chronic viral hepatitis (ICD-10-CM: B18), and unspecified viral hepatitis (ICD-10-CM: B19). Laboratory and clinical parameters comprised body mass index (BMI), alanine aminotransferase (ALT), aspartate aminotransferase (AST), total cholesterol, and triglycerides. Furthermore, participants were categorized according to predefined laboratory thresholds: BMI ≥ 30 kg/m^2^, ALT ≥ 40 U/L, AST ≥ 40 U/L, total cholesterol ≥ 200 mg/dL, and triglycerides ≥ 200 mg/dL.

### 2.5. Statistical Analyses

To account for baseline differences between women with and without cirrhosis, we used propensity score matching (PSM) via the TriNetX platform’s built-in analytical functions. Propensity scores were calculated for each participant using multivariable logistic regression, with the covariates listed in Table 1. Nearest-neighbor matching without replacement was performed using a caliper width of 0.1 standard deviations of the logit of the propensity score. PSM was conducted prior to all subsequent analyses, including both overall and stratified analyses. Covariate balance between the matched groups was assessed using standardized mean differences (SMDs), with an SMD < 0.1 considered indicative of adequate balance between groups [22]. Relative risks (RRs) and risk differences (RDs), along with 95% confidence intervals (CIs), were estimated to compare pregnancy-related outcomes between women with and without cirrhosis. Subgroup analyses were conducted further by age group (18–34 years and 35–49 years). All statistical analyses were performed using the TriNetX Analytics Network Platform.

## 3. Results

### 3.1. Baseline Characteristics of Participants

Table 1 presents the baseline characteristics of the study participants. A total of 2498 pregnant women with cirrhosis and 3,699,378 pregnant women without cirrhosis were included in the study before PSM. Before matching, women in the cirrhosis group were slightly older than those in the non-cirrhosis group (30.4 vs. 28.6 years). The proportion of White participants was higher in the cirrhosis group than in the non-cirrhosis group (61.0% vs. 51.5%), indicating an imbalance in racial distribution prior to matching. In addition, the cirrhosis group had a higher prevalence of lifestyle-related factors, comorbidities, and abnormal laboratory findings compared with the non-cirrhosis group. Specifically, higher proportions of nicotine dependence, alcohol-related disorders, metabolic comorbidities (including diabetes mellitus, hypertension, and hyperlipidemia), liver-related diseases, and abnormal laboratory parameters (including elevated BMI, ALT, and AST) were observed among women with cirrhosis, with several variables showing substantial baseline imbalances (SMD > 0.1).

After PSM, 2485 women remained in each group. The mean age became comparable between the cirrhosis and non-cirrhosis groups (30.4 vs. 30.3 years). The racial distribution, including the proportion of White participants (61.0% vs. 60.8%), was well balanced after matching. Baseline characteristics were well balanced after matching, with all standardized mean differences below 0.1, indicating adequate balance between the matched cohorts.

In addition, the distribution of cirrhosis etiologies based on ICD-10 codes is presented in Supplementary Table S1. Among pregnant women with cirrhosis, the most common diagnosis was other and unspecified cirrhosis of the liver (K74.6), accounting for 37% of cases. This was followed by hepatic fibrosis (K74.0), which accounted for 25%, and alcoholic cirrhosis of the liver (K70.3), representing 11% of the cohort. Other etiologies, including primary biliary cirrhosis, secondary biliary cirrhosis, and hepatic sclerosis, were less frequent.

**Table 1:** Baseline characteristics of pregnant women with versus without cirrhosis before and after propensity score matching.

	Before PSM			After PSM		
	Cirrhosis	Non-Cirrhosis	SMD	Cirrhosis	Non-Cirrhosis	SMD
	(*n* = 2498)	(*n* = 3,699,378)		(*n* = 2485)	(*n* = 2485)	
Age at Index	30.4 ± 6.3	28.6 ± 6.0	0.285	30.4 ± 6.3	30.3 ± 6.2	0.007
Race						
White	1523 (61.0%)	1,903,586 (51.5%)	0.193	1516 (61.0%)	1511 (60.8%)	0.004
Black or African American	331 (13.3%)	632,604 (17.1%)	0.108	329 (13.2%)	335 (13.5%)	0.007
Asian	148 (5.9%)	201,198 (5.4%)	0.021	147 (5.9%)	148 (6.0%)	0.002
Lifestyle						
Nicotine dependence	204 (8.2%)	69,538 (1.9%)	0.291	200 (8.0%)	210 (8.5%)	0.015
Alcohol related disorders	193 (7.7%)	10,265 (0.3%)	0.387	186 (7.5%)	181 (7.3%)	0.008
Comorbidity						
Diabetes mellitus	178 (7.1%)	32,531 (0.9%)	0.323	177 (7.1%)	193 (7.8%)	0.025
Hypertension	191 (7.6%)	44,041 (1.2%)	0.318	188 (7.6%)	196 (7.9%)	0.012
Hyperlipidemia	115 (4.6%)	27,796 (0.8%)	0.24	112 (4.5%)	107 (4.3%)	0.01
Fatty liver	223 (8.9%)	5772 (0.2%)	0.431	220 (8.9%)	236 (9.5%)	0.022
Toxic liver disease	23 (0.9%)	327 (0.0%)	0.134	23 (0.9%)	25 (1.0%)	0.008
Hepatic failure	96 (3.8%)	185 (0.0%)	0.282	88 (3.5%)	55 (2.2%)	0.08
Chronic hepatitis	24 (1.0%)	200 (0.0%)	0.138	22 (0.9%)	17 (0.7%)	0.023
Other inflammatory liver diseases	254 (10.2%)	1299 (0.0%)	0.473	243 (9.8%)	223 (9.0%)	0.028
Other diseases of liver	466 (18.7%)	7935 (0.2%)	0.665	453 (18.2%)	454 (18.3%)	0.001
Chronic viral hepatitis	159 (6.4%)	4033 (0.1%)	0.359	158 (6.4%)	196 (7.9%)	0.06
Unspecified viral hepatitis	101 (4.0%)	4321 (0.1%)	0.278	99 (4.0%)	112 (4.5%)	0.026
Laboratory						
BMI (≥30 kg/m^2^)	531 (21.3%)	351,127 (9.5%)	0.331	526 (21.2%)	551 (22.2%)	0.024
ALT (≥40 U/L)	524 (21.0%)	39,967 (1.1%)	0.67	513 (20.6%)	559 (22.5%)	0.045
AST (≥40 U/L)	498 (19.9%)	26,828 (0.7%)	0.665	486 (19.6%)	514 (20.7%)	0.028
Total cholesterol (≥200 mg/dL)	100 (4.0%)	31,968 (0.9%)	0.205	96 (3.9%)	88 (3.5%)	0.017
Triglyceride (≥200 mg/dL)	67 (2.7%)	8907 (0.2%)	0.205	64 (2.6%)	64 (2.6%)	0.001

Abbreviations: ALT, alanine aminotransferase; AST, aspartate aminotransferase; BMI, body mass index; PSM, propensity score matching; SMD, standardized mean difference.

### 3.2. Risk of Pregnancy-Related Outcomes in Pregnant Women with Cirrhosis

Table 2 presents the risks of pregnancy-related outcomes after PSM. Pregnant women with cirrhosis had a higher risk of several pregnancy-related outcomes compared with those without cirrhosis, including gestational diabetes mellitus (15.5% vs. 11.9%; RR = 1.30; 95% CI, 1.13–1.50, *p* < 0.001; RD = 3.60%; 95% CI, 1.70–5.50), preeclampsia (8.6% vs. 5.7%; RR = 1.52; 95% CI, 1.24–1.87, *p* < 0.001; RD = 2.90%; 95% CI, 1.50–4.40), preterm birth (9.0% vs. 4.9%; RR = 1.85; 95% CI, 1.49–2.29, *p* < 0.001; RD = 4.10%; 95% CI, 2.70–5.60), miscarriage (6.6% vs. 4.8%; RR = 1.38; 95% CI, 1.10–1.73, *p* = 0.005; RD = 1.80%; 95% CI, 0.50–3.10), stillbirth (1.3% vs. 0.5%; RR = 2.67; 95% CI, 1.38–5.17, *p* = 0.004; RD = 0.80%; 95% CI, 0.30–1.30), placental abruption (1.8% vs. 0.8%; RR = 2.20; 95% CI, 1.30–3.72, *p* = 0.003; RD = 1.00%; 95% CI, 0.30–1.60), postpartum hemorrhage (6.9% vs. 4.3%; RR = 1.59; 95% CI, 1.26–2.01, *p* < 0.001; RD = 2.60%; 95% CI, 1.30–3.90), and cesarean delivery (20.0% vs. 17.2%; RR = 1.16; 95% CI, 1.03–1.30, *p* = 0.012; RD = 2.80%; 95% CI, 0.60–4.90).

No statistically significant differences were observed for premature rupture of membranes (7.5% vs. 7.1%; RR = 1.06; 95% CI, 0.87–1.30, *p* = 0.570; RD = 0.40%; 95% CI, −1.00–1.90) or dystocia (9.1% vs. 8.4%; RR = 1.08; 95% CI, 0.90–1.29, *p* = 0.402; RD = 0.70%; 95% CI, −0.90–2.30).

### 3.3. Age-Stratified Risk of Pregnancy-Related Outcomes in Pregnant Women with Cirrhosis

Table 3 presents the age-stratified risk of pregnancy-related outcomes after PSM. In the age-stratified analysis among women aged 18–34 years, cirrhosis was associated with a higher risk of multiple pregnancy-related outcomes after PSM, including gestational diabetes mellitus, preeclampsia, preterm birth, miscarriage, stillbirth, placental abruption, postpartum hemorrhage, and cesarean delivery. No statistically significant differences were observed for premature rupture of membranes or dystocia in this age group.

Among women aged 35–49 years, no statistically significant differences were observed between the cirrhosis and non-cirrhosis groups for pregnancy-related outcomes. Nevertheless, most outcomes showed a consistent trend toward increased risk among women with cirrhosis.

## 4. Discussion

Before PSM, there were significant differences in age, race, lifestyle factors, comorbidities, and laboratory profiles between pregnant women with and without cirrhosis. These differences were balanced after matching, which allowed a reliable comparison of outcomes. After PSM analyses revealed that cirrhosis was independently associated with significantly increased risks of several adverse pregnancy outcomes, including gestational diabetes, preeclampsia, preterm birth, miscarriage, stillbirth, placental abruption, PPH, and cesarean delivery. Additionally, age-stratified analysis demonstrated that the increased risk persisted in the younger age group (18–34 years). However, older women (35–49 years) did not show a significant risk of adverse pregnancy outcomes. Taken together, our findings highlight the substantial maternal and fetal risks posed by cirrhosis during pregnancy, especially in the younger age group.

Pregnancy in women with liver cirrhosis is rare, with an estimated incidence of 1 in 3333 to 5950 pregnancies [23,24]. However, the incidence of pregnancy with cirrhosis has more than doubled in recent years [25]. Despite improvements in pregnancy outcomes, cirrhosis remains associated with increased risks of adverse maternal and fetal outcomes [24,25]. Potential complications of pregnancy with adverse outcomes include preterm delivery, postpartum hemorrhage, hypertensive disorders, and gastrointestinal bleeding [25]. Nonetheless, maternal mortality is currently rare, and live birth rates are high [24,25]. Our study also identified an overall incidence of 0.06% in the total study cohort.

In our study, the median age of women with and without cirrhosis was 30.4 and 30.3 years, respectively. Previous studies have indicated that the median age of conception among women with cirrhosis was 26 years [26]. Although live birth rates are similar between women with and without cirrhosis, women with cirrhosis are less likely to reach full term [26]. Nonetheless, most pregnancies in women with cirrhosis have successful outcomes despite these risks; however, maternal and fetal complication rates remain high among patients with decompensated cirrhosis [27].

Although pregnancy with cirrhosis is associated with increased risks of adverse outcomes, including caesarean delivery, low birth weight, and preterm delivery [19,24], severe maternal and fetal complications are rare. Additionally, chronic liver disease, including cirrhosis, has been associated with a 2.6-fold increased risk of preeclampsia [28]. However, other studies have reported that pregnancy with cirrhosis is not associated with increased rates of gestational diabetes or preeclampsia [19]. Our study demonstrated that pregnancy complicated by liver cirrhosis is associated with an increased risk of most adverse pregnancy outcomes.

Pregnancy with liver cirrhosis involves significant challenges, especially when considering the mode of delivery [5]. These patients often require cesarean section due to the increased risk of cirrhosis-related complications, including portal hypertension and esophageal varices, which can lead to life-threatening bleeding during vaginal delivery [19]. Compared with women without cirrhosis, those with liver cirrhosis have a higher incidence of cesarean sections, with the reported rates reaching as high as 73.6% [3]. Additionally, the presence of esophageal varices, which is a common complication of cirrhosis, often necessitates cesarean section to prevent bleeding during labor [27]. However, cesarean sections in patients with cirrhosis are associated with increased risks of postpartum hemorrhage and other complications, including massive ascites, which require careful postoperative management [1]. Further, anesthetic management during cesarean section is complex, given the altered portal hemodynamics and risk of variceal hemorrhage, which require specialized care [29]. In our study, pregnancy with cirrhosis had a higher risk for cesarean section (RR: 1.16).

Our findings are broadly consistent with, yet provide important nuances beyond, the landmark meta-analysis by van der Slink et al. (2022) [1], which pooled 2912 pregnancies across 11 studies and demonstrated that women with liver cirrhosis faced significantly elevated risks of preterm delivery (OR 6.7, 95% CI 5.1–9.1), caesarean section (OR 2.6, 95% CI 1.7–3.9), preeclampsia (OR 3.8, 95% CI 2.2–6.5), and small-for-gestational-age neonates (OR 2.6, 95% CI 1.6–4.2). In our propensity score-matched cohort, the direction of risk elevation was confirmed across these same outcomes; however, the magnitude of effect estimates was comparatively more modest, with preterm birth demonstrating an RR of 1.85 (95% CI 1.49–2.29) and preeclampsia an RR of 1.52 (95% CI 1.24–1.87), and cesarean delivery an RR of 1.16 (95% CI 1.03–1.30). This attenuation likely reflects methodological differences, including the use of propensity score matching in our study to control for confounders such as comorbidities and demographic characteristics, as well as potential differences in the underlying patient populations and the more contemporary time period captured in our cohort, during which advances in multidisciplinary antenatal care may have contributed to improved outcomes. Importantly, our study extends the existing evidence by additionally quantifying risks for outcomes not addressed in the prior meta-analysis, including gestational diabetes mellitus (RR 1.30, 95% CI 1.13–1.50), miscarriage (RR 1.38, 95% CI 1.10–1.73), stillbirth (RR 2.67, 95% CI 1.38–5.17), placental abruption (RR 2.20, 95% CI 1.30–3.72), and postpartum hemorrhage (RR 1.59, 95% CI 1.26–2.01), thereby providing a more comprehensive characterization of the obstetric risk profile associated with cirrhosis. Notably, premature rupture of membranes and dystocia did not reach statistical significance in our analysis (RR 1.06 and 1.08, respectively), suggesting that not all obstetric complications are uniformly elevated in this population. Taken together, our results reinforce and expand upon the conclusions of van der Slink et al. (2022) [1], affirming that cirrhosis confers a broad and clinically meaningful increase in pregnancy-related morbidity while highlighting the value of contemporary, confounder-adjusted analyses in refining risk estimates for counseling and clinical decision-making.

Our findings are in agreement with several prior cohort studies that have consistently documented elevated obstetric and perinatal risks in pregnant women with liver cirrhosis. Gao et al. (2021) [3], in a case–control study of 97 continued pregnancies complicated by cirrhosis, reported notably high rates of cesarean section (73.6%), postpartum hemorrhage (13.8%), and low birth weight infants (13.6%), with severe maternal adverse events occurring in 32.0% of cases and higher CTP scores identified as the principal predictor of poor outcomes. Similarly, Tan et al. (2024) [2] described a preterm birth incidence of 46.51% among 43 cirrhotic pregnancies in a Chinese university hospital cohort, alongside increased risks of cesarean section, intrahepatic cholestasis of pregnancy, thrombocytopenia, and postpartum hemorrhage, with intrahepatic cholestasis of pregnancy and total bilirubin emerging as key predictors of preterm birth. More recently, Nana et al. (2025) [8], utilizing the UK Obstetric Surveillance System in a prospective national cohort of 52 cases, reported a preterm birth rate of 51.2% and demonstrated that the ALBI score predicted maternal decompensation, ICU admission, and preterm birth, while untreated varices were associated with significantly higher rates of variceal hemorrhage. While the direction of risk across these studies is consistently aligned with our own results—confirming elevated rates of preterm birth, postpartum hemorrhage, preeclampsia, and cesarean delivery in cirrhotic pregnancies—the absolute risk estimates reported in prior single-center and national cohort studies tend to be considerably higher than those observed in our propensity score-matched analysis, where preterm birth occurred in 9.0% and postpartum hemorrhage in 6.9% of cirrhotic pregnancies. This discrepancy likely reflects differences in study design, patient selection, disease severity at enrollment, and the effectiveness of propensity score matching in our study in minimizing confounding by indication, rather than a true divergence in the underlying biology of cirrhosis-associated pregnancy risk. Collectively, these studies, alongside our own data, reinforce the consistent message that cirrhosis meaningfully elevates the risk of adverse maternal and fetal outcomes, while highlighting the importance of methodological rigor and population-level analyses in generating risk estimates that are applicable to contemporary clinical practice.

Our age-stratified analysis revealed that the elevated risks associated with cirrhosis in pregnancy were more pronounced and statistically significant among younger women aged 18–34 years compared to those aged 35–49 years, a finding that warrants contextual comparison with existing literature. In the younger age group, statistically significant elevations were observed across a broad range of outcomes including preterm birth (RR 1.66, 95% CI 1.32–2.09), preeclampsia (RR 1.39, 95% CI 1.10–1.76), gestational diabetes mellitus (RR 1.26, 95% CI 1.07–1.48), miscarriage (RR 1.46, 95% CI 1.09–1.95), postpartum hemorrhage (RR 1.51, 95% CI 1.15–1.97), stillbirth (RR 2.40, 95% CI 1.15–5.00), placental abruption (RR 2.31, 95% CI 1.29–4.14), and cesarean delivery (RR 1.17, 95% CI 1.02–1.34), whereas in the older age group (35–49 years), none of these associations reached statistical significance. This pattern is of particular clinical relevance given that the majority of pregnancies complicated by cirrhosis in prior studies have similarly occurred predominantly in younger women of reproductive age. Gao et al. (2021) [3] did not perform age-stratified analyses but reported high overall rates of severe adverse maternal events (32.0%) in a cohort whose cirrhotic pregnancies were managed at a single center, with CTP score—rather than maternal age—identified as the primary predictor of adverse outcomes, suggesting that disease severity may modulate risk more powerfully than age in populations with more advanced hepatic dysfunction. Tan et al. (2024) [2] likewise did not stratify by age but observed a preterm birth incidence of 46.51% in their cirrhotic cohort, a figure considerably higher than the 3.90 percentage point absolute risk difference we observed in the 18–34 age group, which may reflect the more severe disease profile and smaller sample size of that single-center cohort. Nana et al. (2025) [8] similarly lacked age-stratified data but demonstrated that hepatic reserve, as captured by the ALBI score, was the dominant predictor of preterm birth and maternal decompensation, indirectly supporting the interpretation that in older women with cirrhosis, where competing comorbidities and advanced disease may be more prevalent, the relative contribution of cirrhosis per se to obstetric risk becomes more difficult to isolate in propensity score-matched comparisons. The attenuation of statistically significant risk elevations in the 35–49 age group in our study may therefore reflect a combination of reduced statistical power due to smaller subgroup sample sizes, greater heterogeneity in the underlying etiology and severity of cirrhosis in older patients, and the increased baseline obstetric risk inherent to advanced maternal age in the comparator group, which may narrow the relative risk differential. Taken together, our age-stratified findings add an important dimension to the existing literature by suggesting that younger women with cirrhosis represent a particularly vulnerable subgroup in whom targeted antenatal surveillance and early intervention may yield the greatest clinical benefit.

From a clinical perspective, the management of pregnancy complicated by preexisting liver disease demands proactive, individualized care anchored in a multidisciplinary framework. Hepatologists, obstetricians, neonatologists, and—where relevant—transplant specialists must collaborate from the pre-conception stage through to the postpartum period, as complications can arise at any point along this continuum [4,5]. Pre-conception counseling represents the first and perhaps most critical intervention, allowing clinicians to optimize disease control, review medication safety, and counsel patients on realistic risks before pregnancy is established; this is particularly well-illustrated in AIH, where achieving complete biochemical response prior to conception significantly reduces the likelihood of gestational relapse and hypertensive complications [11,12]. During pregnancy, risk stratification tools such as the CTP score, ALBI score, and noninvasive fibrosis markers including FIB-4 and APRI provide clinicians with objective parameters to identify patients at highest risk of hepatic decompensation, preterm birth, and ICU admission, enabling timely escalation of care [8,9,30]. Variceal screening and the judicious use of non-selective beta-blockers for portal hypertension prophylaxis should be incorporated into antenatal care, as untreated varices are associated with significantly higher rates of hemorrhagic complications, while available evidence suggests that beta-blockers are well tolerated in this population without adverse fetal effects [6,31]. In the postpartum period, vigilance must be maintained, as hepatic decompensation and AIH relapse are disproportionately common in the weeks following delivery, representing a window of vulnerability that is frequently underappreciated in clinical practice [8,11]. Ultimately, while pregnancy in women with chronic liver disease carries meaningful risks, evidence increasingly supports that with appropriate counseling, monitoring, and timely intervention, favorable maternal and fetal outcomes are achievable across a broad range of underlying hepatic conditions [4,10].

### Strengths and Limitations

This study leveraged a large dataset comprising >3.6 million pregnancies, providing robust statistical power to evaluate rare exposures and outcomes. The use of PSM enabled well-balanced comparison groups, minimizing confounding bias and enhancing the validity of observed associations. Additionally, we performed a comprehensive assessment of detailed baseline characteristics, including demographic, lifestyle, comorbidity, and laboratory variables, which facilitated nuanced control of potential confounders. Moreover, age-stratified analyses provided further insights into differences in cirrhosis-related pregnancy risks across maternal age groups, improving the generalizability and clinical relevance of the findings.

However, this study has several limitations. The retrospective study design may have allowed residual confounding from unmeasured variables, including the severity and etiology of cirrhosis, socioeconomic status, medication use, and access to prenatal care. The lack of longitudinal follow-up limits insights into long-term maternal and neonatal outcomes. A major limitation of this study is the lack of detailed clinical information regarding the severity of cirrhosis. Important parameters such as Child–Pugh class, MELD score, and complications related to portal hypertension were not consistently available in the TriNetX database. Because pregnancy outcomes are strongly influenced by the severity of liver disease, the inability to stratify patients by disease severity may introduce heterogeneity within the cirrhosis cohort and limit the clinical interpretation of the findings. The observed risks may therefore represent an average effect across a spectrum of disease severity and could potentially underestimate or overestimate the true risks in patients with compensated or decompensated cirrhosis. The age-stratified analysis showed that women aged 35–49 years did not demonstrate statistically significant differences in several outcomes. This finding may be explained by the relatively smaller sample size in this subgroup, which may limit statistical power. In addition, women with cirrhosis who become pregnant at older ages may represent a selected population with relatively stable disease and closer clinical monitoring, potentially contributing to better outcomes. Residual confounding due to unavailable clinical variables may also partly explain this observation. Diagnoses and outcomes were identified using ICD codes, which may introduce misclassification bias due to coding inaccuracies or variability in clinical documentation. The TriNetX network primarily includes data from participating healthcare organizations, which may introduce selection bias and limit the generalizability of the findings to populations outside these healthcare systems. Certain clinical encounters occurring outside participating institutions, particularly outpatient visits, may not be fully captured in the database, potentially resulting in incomplete clinical information. Although demographic information is available, the granularity of ethnicity data in the database is limited, which may restrict the ability to evaluate potential differences across detailed ethnic subgroups. Another limitation is that the TriNetX platform does not allow reliable linkage between maternal and neonatal records. Therefore, neonatal outcomes such as low birth weight, NICU admission, neonatal mortality, and congenital anomalies could not be assessed.

## 5. Conclusions

Our findings suggested that pregnant women with liver cirrhosis are associated with higher risks of several adverse maternal and fetal outcomes, including preterm birth, metabolic, hypertensive, hemorrhagic complications, and cesarean delivery. These associations appeared more evident in younger women but were less pronounced in the older age group. These findings underscore the importance of careful clinical monitoring and multidisciplinary care for pregnant women with cirrhosis. It may help inform risk assessment and perinatal management strategies aimed at improving maternal and neonatal outcomes.

## Figures and Tables

**Table 2:** Risk of pregnancy-related outcomes in pregnant women with versus without cirrhosis after propensity score matching.

Outcome	Patients with Outcome		Relative Risk (95% CI)	*p*-Value ^a^	Risk Difference (95% CI) ^b^
	Cirrhosis (%)	Non-Cirrhosis (%)			
Gestational diabetes mellitus	386 (15.5%)	297 (11.9%)	1.30 (1.13, 1.50)	<0.001	3.60 (1.70, 5.50)
Preeclampsia	213 (8.6%)	140 (5.7%)	1.52 (1.24, 1.87)	<0.001	2.90 (1.50, 4.40)
Premature rupture of membranes	186 (7.5%)	175 (7.1%)	1.06 (0.87, 1.30)	0.570	0.40 (−1.00, 1.90)
Preterm birth	224 (9.0%)	121 (4.9%)	1.85 (1.49, 2.29)	<0.001	4.10 (2.70, 5.60)
Miscarriage	164 (6.6%)	119 (4.8%)	1.38 (1.10, 1.73)	0.005	1.80 (0.50, 3.10)
Stillbirth	32 (1.3%)	12 (0.5%)	2.67 (1.38, 5.17)	0.004	0.80 (0.30, 1.30)
Placental abruption	44 (1.8%)	20 (0.8%)	2.20 (1.30, 3.72)	0.003	1.00 (0.30, 1.60)
Dystocia	226 (9.1%)	209 (8.4%)	1.08 (0.90, 1.29)	0.402	0.70 (−0.90, 2.30)
Postpartum hemorrhage	172 (6.9%)	108 (4.3%)	1.59 (1.26, 2.01)	<0.001	2.60 (1.30, 3.90)
Cesarean delivery	498 (20.0%)	429 (17.2%)	1.16 (1.03, 1.30)	0.012	2.80 (0.60, 4.90)

Abbreviations: CI, confidence interval. *p*-value < 0.05 are presented in bold. ^a^: *p*-value for relative risk. ^b^: Risk differences are expressed in percentage points.

**Table 3:** Age-stratified risk of pregnancy-related outcomes in pregnant women with versus without cirrhosis after propensity score matching.

Outcome	Age Group (18–34 Years)			Age Group (35–49 Years)		
	Relative Risk (95% CI)	*p*-Value ^a^	Risk Difference (95% CI) ^b^	Relative Risk (95% CI)	*p*-value ^a^	Risk Difference (95% CI) ^b^
Gestational diabetes mellitus	1.26 (1.07, 1.48)	0.005	3.20 (0.90, 5.40)	1.05 (0.81, 1.37)	0.716	0.80 (−3.20, 4.80)
Preeclampsia	1.39 (1.10, 1.76)	0.006	2.40 (0.70, 4.10)	1.38 (0.92, 2.07)	0.119	2.30 (−0.60, 5.10)
Premature rupture of membranes	1.15 (0.91, 1.45)	0.240	1.00 (−0.70, 2.70)	1.35 (0.86, 2.13)	0.195	1.80 (−0.80, 4.40)
Preterm birth	1.66 (1.32, 2.09)	<0.001	3.90 (2.10, 5.60)	1.38 (0.90, 2.12)	0.141	2.10 (−0.70, 4.90)
Miscarriage	1.46 (1.09, 1.95)	0.011	1.90 (0.50, 3.30)	1.27 (0.86, 1.88)	0.231	1.80 (−1.20, 4.70)
Stillbirth	2.40 (1.15, 5.00)	0.020	0.80 (0.10, 1.40)	-	-	-
Placental abruption	2.31 (1.29, 4.14)	0.005	1.20 (0.40, 1.90)	-	-	-
Dystocia	0.95 (0.78, 1.16)	0.612	−0.50 (−2.40, 1.40)	0.98 (0.67, 1.44)	0.918	−0.20 (−3.20, 2.80)
Postpartum hemorrhage	1.51 (1.15, 1.97)	0.003	2.40 (0.80, 3.90)	1.29 (0.82, 2.03)	0.271	1.50 (−1.10, 4.10)
Cesarean delivery	1.17 (1.02, 1.34)	0.024	2.90 (0.40, 5.40)	1.00 (0.80, 1.25)	1.000	0.00 (−4.50, 4.50)

Abbreviations: CI, confidence interval. TriNetX does not report exact patient counts when sample sizes are too small, to protect patient privacy. *p*-value < 0.05 are presented in bold. ^a^: *p*-value for relative risk. ^b^: Risk differences are expressed in percentage points.

## Data Availability

Due to data protection regulations, sharing the information in this article is not permissible.

## Supplementary Materials

The following supporting information can be downloaded at: https://www.mdpi.com/article/10.3390/medicina62030591/s1, Table S1: Distribution of cirrhosis diagnoses based on ICD-10 codes in the study cohort.

## Abbreviations

The following abbreviations are used in this manuscript:

EHR	electronic health record
HCOs	healthcare organizations
ICD-10-CM	International Classification of Diseases, Tenth Revision, Clinical Modification
CPT	Current Procedural Terminology
BMI	body mass index
AST	aspartate aminotransferase
ALT	alanine aminotransferase
PSM	propensity score matching
SMDs	standardized mean differences
