# Supplementary material for: Impact of Liver Cirrhosis on Pregnancy Outcomes: A Retrospective Cohort Study from the TriNetX Global Collaborative Network
Source: Medicina (Kaunas). 2026 Mar 20;62(3):591. doi: 10.3390/medicina62030591 (PMC13028217; doi:10.3390/medicina62030591)
Supplement: Supplementary file 1 [file medicina-62-00591-s001.zip › medicina-4172601-supplementary.pdf]

Supplementary Table S1. Distribution of cirrhosis diagnoses based on ICD-10 codes in the study cohort.

ICD-10-CM	Diagnosis	Patients n (%)
K74.0	Hepatic fibrosis	625 (25%)
K74.1	Hepatic sclerosis	100 (4%)
K74.3	Primary biliary cirrhosis	200 (8%)
K74.4	Secondary biliary cirrhosis	75 (3%)
K74.5	Biliary cirrhosis, unspecified	100 (4%)
K74.6	Other and unspecified cirrhosis of liver	924 (37%)
K70.3	Alcoholic cirrhosis of liver	275 (11%)

ICD-10-CM: International Classification of Diseases, Tenth Revision, Clinical Modification.
